# Supplementary material for: Understanding Magnetization Dynamics of a Magnetic Nanoparticle with a Disordered Shell Using Micromagnetic Simulations
Source: Nanomaterials (Basel). 2020 Jun 11;10(6):1149. doi: 10.3390/nano10061149 (PMC7353241; doi:10.3390/nano10061149)
Supplement: Supplementary file 1 [file nanomaterials-10-01149-s001.pdf]

Supplementary Materials

Understanding Magnetization Dynamics of a Magnetic Nanoparticle with a Disordered Shell Using Micromagnetic Simulations

David Aurélio and Jana Vejpravová *

Department of Condensed Matter Physics, Faculty of Mathematics and Physics, Charles University, Ke Karlovu 5, 121 16 Prague 2, Czech Republic; davidaurelio@mag.mff.cuni.cz

* Correspondence: jana@mag.mff.cuni.cz

1. Simulated Magnetization Isotherms and Visualization of Magnetization Reversal of a Single Magnetic Nanoparticle with a Core-Shell Structure

The Supplementary Information (SI) presents individual magnetization isotherms and 3D images of the spin structure during the magnetization reversal at different temperatures for all the different shell thicknesses of the simulated 5.5 nm core diameter nanoparticle (NP). The color code used in the 3D images has the following meaning: cyan—spins are parallel with respect to the applied field and the coordinate z-axis (magnetizing the sample in a positive magnetic field), red—spins are parallel with respect to the applied field and antiparallel to the coordinate z-axis (magnetizing the sample in a negative magnetic field).

1.1. Magnetization Reversal for a Shell Thickness of 0.5 nm.

In this part, results on the simulations of a NP consisting of a 5.5. nm core and 0.5 nm thick shell (6.5 nm in diameter) are summarized.

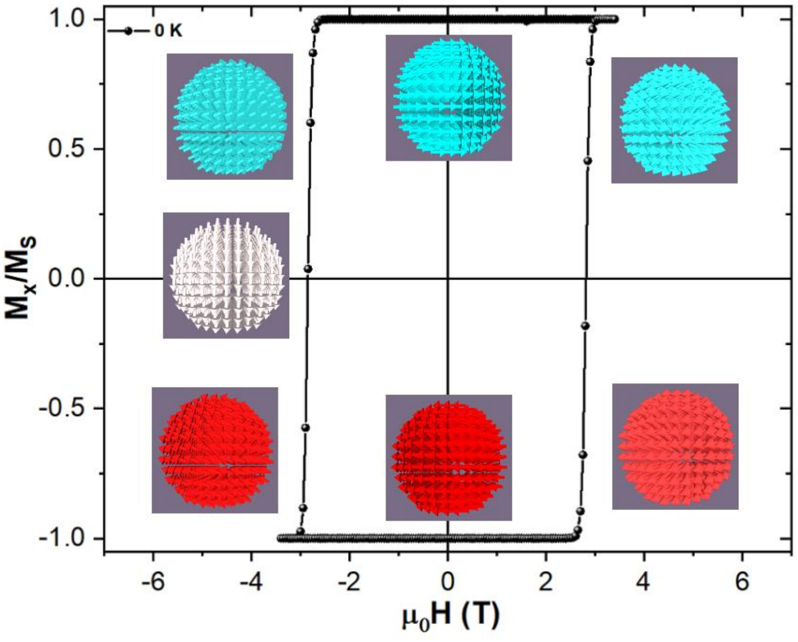

Figure S1. Simulation of the magnetization reversal with the thermal field setting at 0 K. The insets show the 3D spin structure of the NP, during the magnetization cycle, as the external magnetic field sweeps from the positive to negative direction and back. It is possible to see that at 0 K the transition is quite smooth and represents well the typical single domain NP behavior in the blocked state.

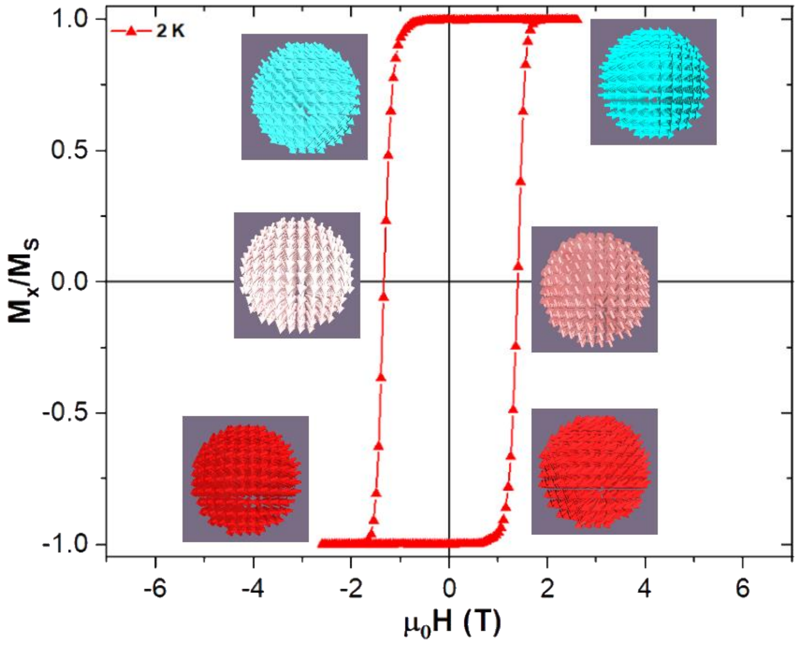

Figure S2. Simulation of the magnetization reversal with the thermal field setting at 2 K. The insets show the 3D spin structure of the NP, during the magnetization cycle, as the external magnetic field sweeps from the positive to negative direction and back. Now at 2 K the transition becomes slightly less smooth, but still represents well the typical single domain NP behavior in the blocked state.

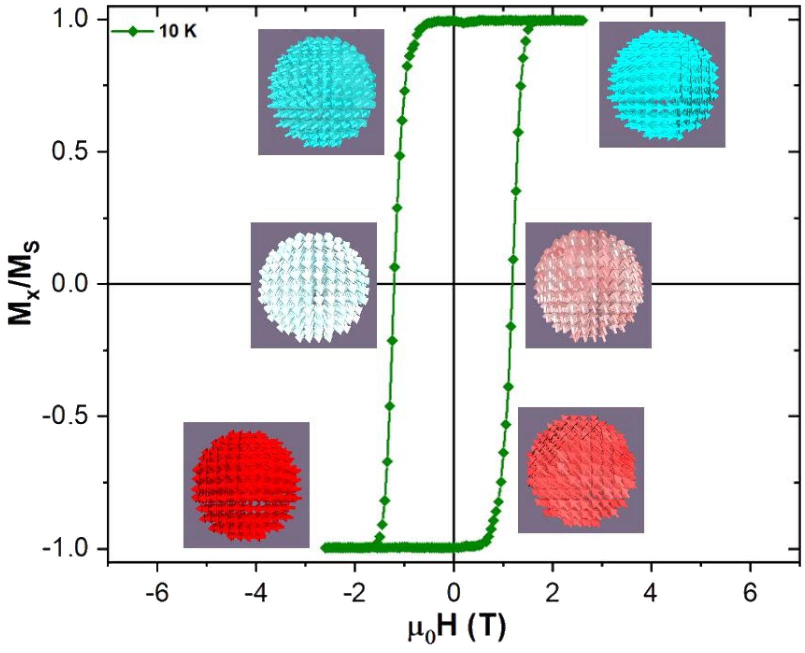

Figure S3. Simulation of the magnetization reversal with the thermal field setting at 10 K. The insets show the 3D spin structure of the NP, during the magnetization cycle, as the external magnetic field sweeps from the positive to negative direction and back. At 10 K the transition continues to be mostly smooth, with some more oscillations of the average magnetization, in particular when the reversal starts. Nonetheless the exchange interaction is still the most relevant, keeping the first neighboring spins quite parallel.

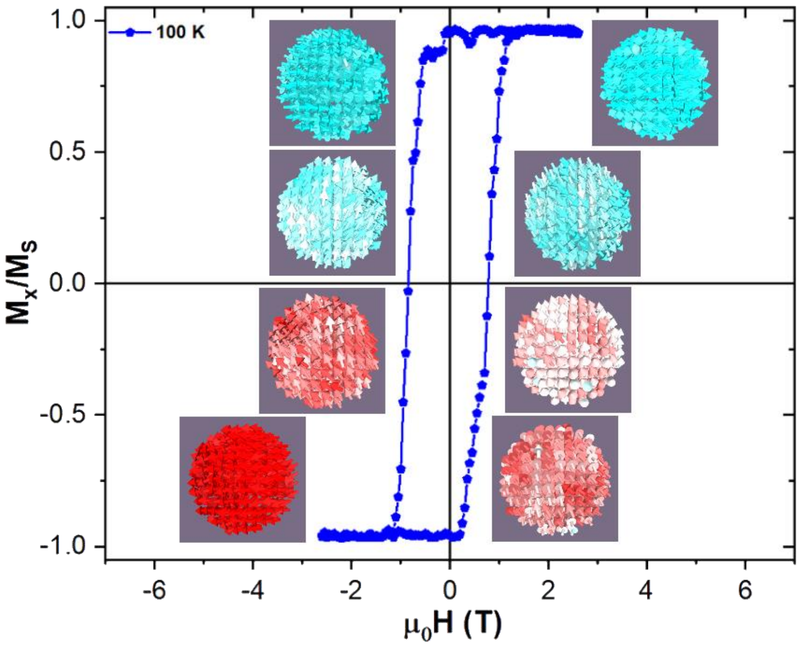

Figure S4. Simulation of the magnetization reversal with the thermal field setting at 100 K. The insets show the 3D spin structure of the NP, during the magnetization cycle, as the external magnetic field sweeps from the positive to negative direction and back. Now at 100 K the transition is no longer very smooth, with several oscillations of the average magnetization before and during the reversal process. The exchange interaction starts to compete more relevantly with the thermal agitation introduced by the thermal field. First neighboring spins start to struggle to be very parallel between each other, but still the overall behavior of the NP is that of single domain.

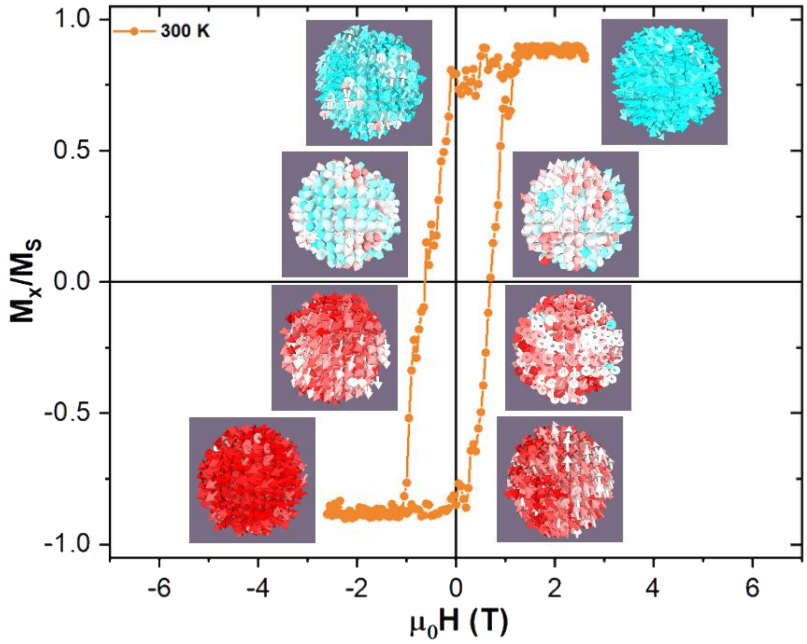

Figure S5. Simulation of the magnetization reversal with the thermal field setting at 300 K. The insets show the 3D spin structure of the NP, during the magnetization cycle, as the external magnetic field sweeps from the positive to negative direction and back. At the room temperature of 300 K the transition is not smooth, with many oscillations of the average magnetization and individual spins vibrations throughout the entire reversal process. The exchange interaction competes actively with the thermal agitation introduced by the thermal field. First neighboring spins struggle to be very parallel between each other, nonetheless the behavior of the NP magnetization dynamics is that of the expected single domain particle.

1.2. Magnetization Reversal for a Shell Thickness of 2.0 nm.

In this part, results on the simulations of a NP consisting of a 5.5 nm core and 2 nm thick shell (9.5 nm in diameter) are summarized. Its individual spin dynamics is quite similar to that seen before for both the 0.5 nm shell thickness. The main differences residing on the coercivity, has it is discussed in the main text of the paper.

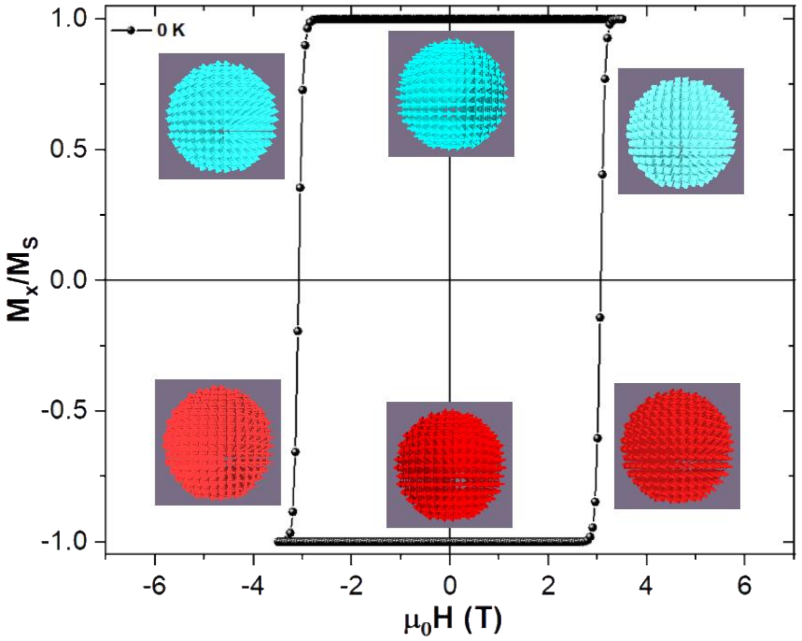

Figure S6. Simulation of the magnetization reversal with the thermal field setting at 0 K. The insets show the 3D spin structure of the NP, during the magnetization cycle, as the external magnetic field sweeps from the positive to negative direction and back. It is possible to see that at 0 K the transition is quite smooth, where the exchange interaction is the most relevant, keeping the first neighboring spins quite parallel and thus a good representation of the typical single domain NP behavior in the blocked state.

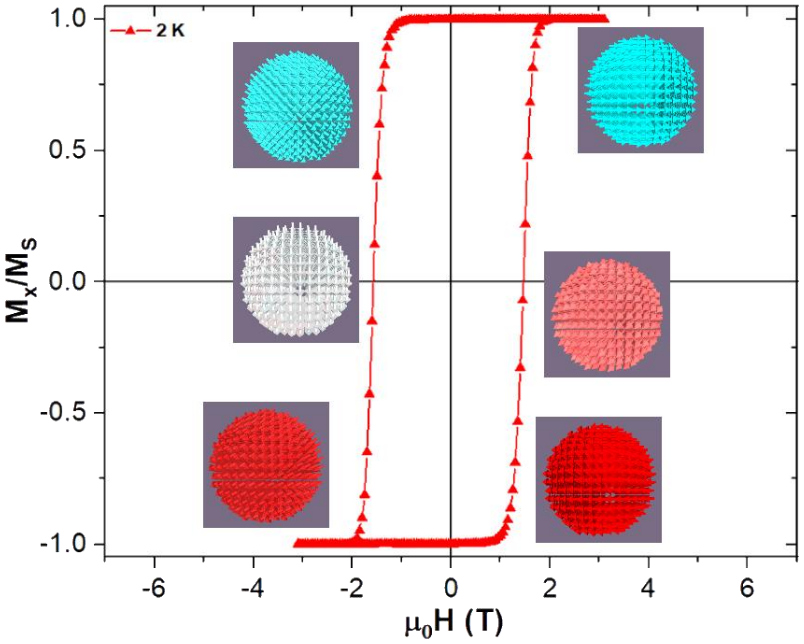

Figure S7. Simulation of the magnetization reversal with the thermal field setting at 2 K. The insets show the 3D spin structure of the NP, during the magnetization cycle, as the external magnetic field sweeps from the positive to negative direction and back. Now at 2 K the transition becomes slightly less smooth, but still the exchange interaction is the most relevant, keeping the first neighboring spins quite parallel and thus a good representation of the typical single domain NP behavior in the blocked state.

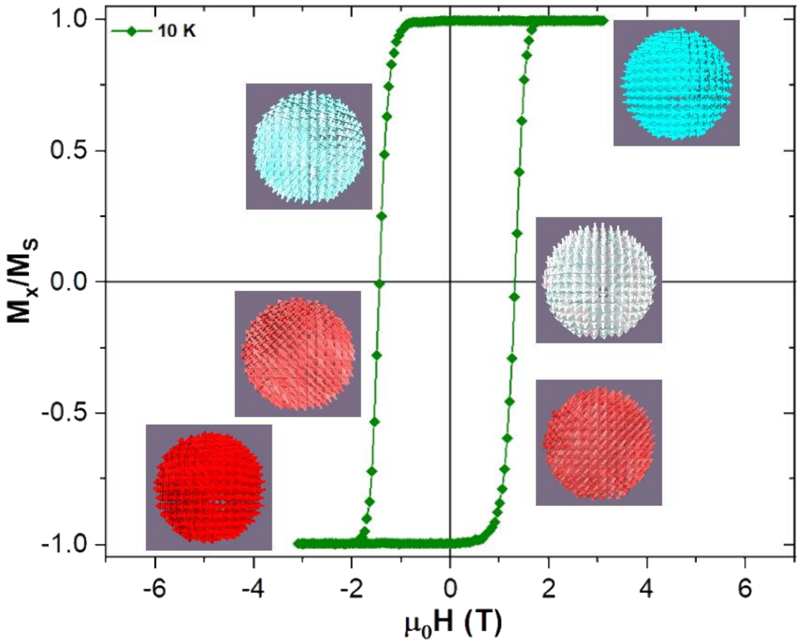

Figure S8. Simulation of the magnetization reversal with the thermal field setting at 10 K. The insets show the 3D spin structure of the NP, during the magnetization cycle, as the external magnetic field sweeps from the positive to negative direction and back. At 10 K the transition continues to be mostly smooth, with some more oscillations of the average magnetization, in particular when the reversal starts. Nonetheless the exchange interaction is still the most relevant, keeping the first neighboring spins quite parallel and thus a good representation of the typical single domain NP behavior in the blocked state.

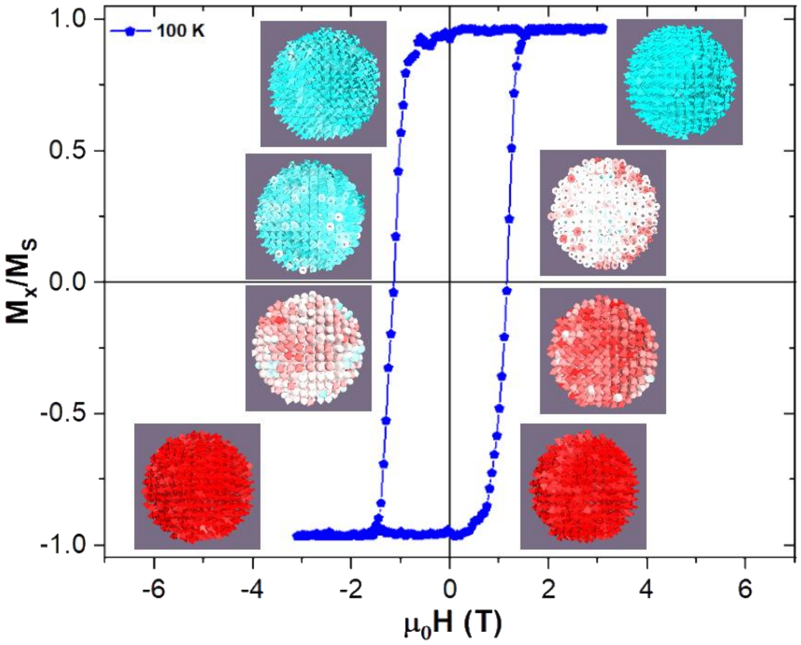

Figure S9. Simulation of the magnetization reversal with the thermal field setting at 100 K. The insets show the 3D spin structure of the NP, during the magnetization cycle, as the external magnetic field sweeps from the positive to negative direction and back. Now at 100 K the transition is no longer very smooth, with several oscillations of the average magnetization before and during the reversal process. The exchange interaction starts to compete more relevantly with the thermal agitation introduced by the thermal field. First neighboring spins start to struggle to be very parallel between each other, but still the overall behavior of the NP is that of single domain in the blocked state.

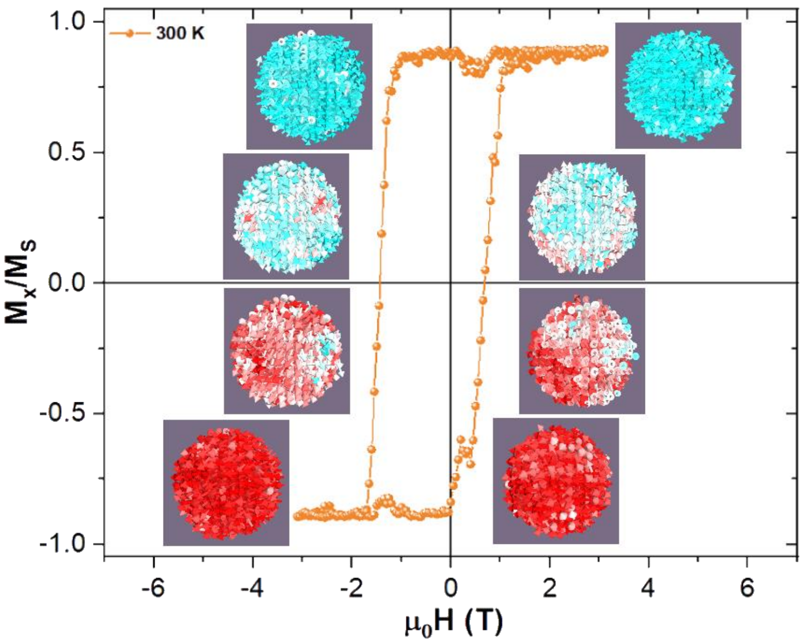

Figure S10. Simulation of the magnetization reversal with the thermal field setting at 300 K. The insets show the 3D spin structure of the NP, during the magnetization cycle, as the external magnetic field sweeps from the positive to negative direction and back. At the room temperature of 300 K the transition is not smooth, with many oscillations of the average magnetization and individual spins fluctuations throughout the entire reversal process. The exchange interaction competes actively with the thermal agitation introduced by the thermal field. First neighboring spins struggle to be very parallel between each other.

1.3. Magnetization Reversal for a Shell Thickness of 3.0 nm.

In this part, results on the simulations of a NP consisting of a 5.5 nm core and 3 nm thick shell (11.5 nm in diameter) are summarized. Its individual spin dynamics is quite similar to that seen before for both the 0.5 nm and 2.0 nm shell thicknesses. The main differences residing on the coercivity, has it is discussed in the main text of the paper.

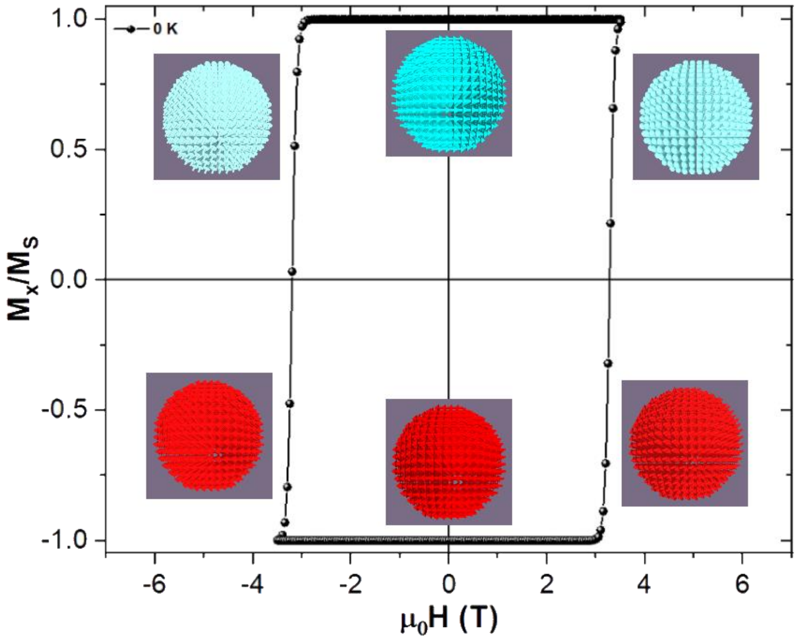

Figure S11. Simulation of the magnetization reversal with the thermal field setting at 0 K. The insets show the 3D spin structure of the NP, during the magnetization cycle, as the external magnetic field sweeps from the positive to negative direction and back. It is possible to see that at 0 K the transition is quite smooth, where the exchange interaction is the most relevant, keeping the first neighboring spins quite parallel and thus a good representation of the typical single domain NP behavior.

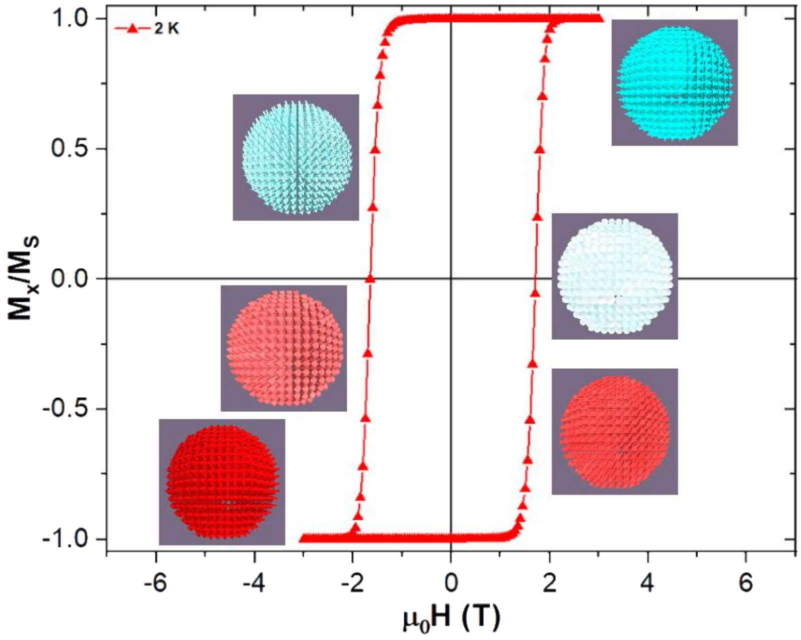

Figure S12. Simulation of the magnetization reversal with the thermal field setting at 2 K. The insets show the 3D spin structure of the NP, during the magnetization cycle, as the external magnetic field sweeps from the positive to negative direction and back. Now at 2 K the transition becomes slightly less smooth, but still the exchange interaction is the most relevant, keeping the first neighboring spins quite parallel and thus a good representation of the typical single domain NP behavior.

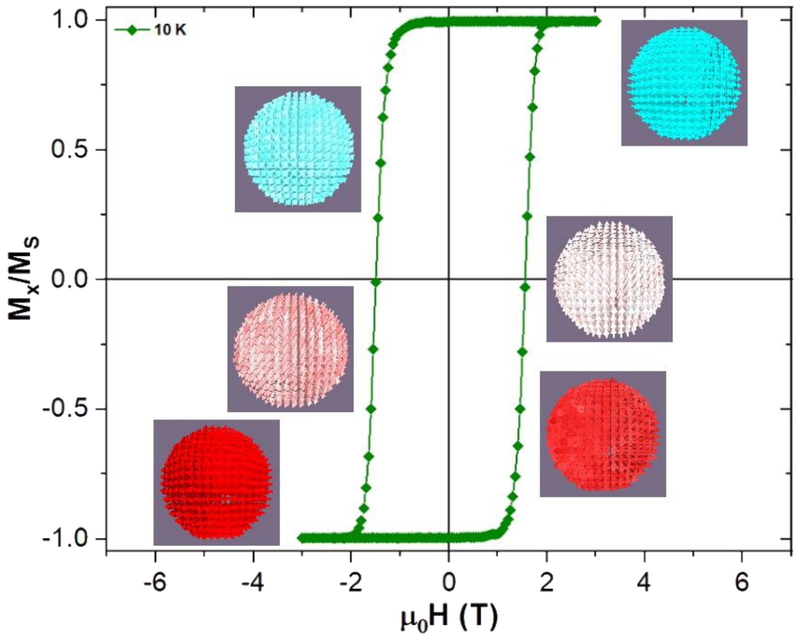

Figure S13. Simulation of the magnetization reversal with the thermal field setting at 10 K. The insets show the 3D spin structure of the NP, during the magnetization cycle, as the external magnetic field sweeps from the positive to negative direction and back. At 10 K the transition continues to be mostly smooth, with some more oscillations of the average magnetization, in particular when the reversal starts. Nonetheless the exchange interaction is still the most relevant, keeping the first neighboring spins quite parallel and thus a good representation of the typical single domain NP behavior.

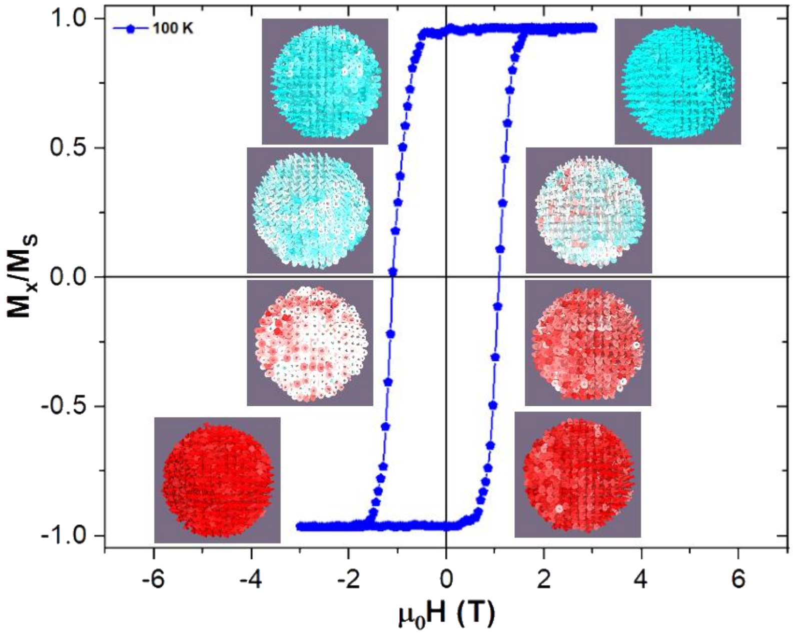

Figure S14. Simulation of the magnetization reversal with the thermal field setting at 100 K. The insets show the 3D spin structure of the NP, during the magnetization cycle, as the external magnetic field sweeps from the positive to negative direction and back. Now at 100 K the transition is no longer very smooth, with several oscillations of the average magnetization before and during the reversal process. The exchange interaction starts to compete more relevantly with the thermal agitation introduced by the thermal field. First neighboring spins start to struggle to be very parallel between each other, but still the overall behavior of the NP is that of single domain.

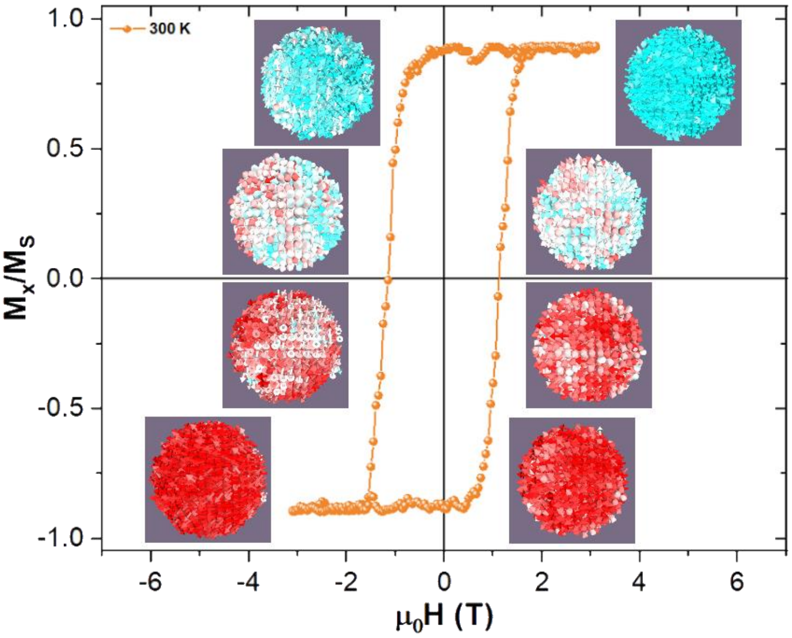

Figure S15. Simulation of the magnetization reversal with the thermal field setting at 300 K. The insets show the 3D spin structure of the NP, during the magnetization cycle, as the external magnetic field sweeps from the positive to negative direction and back. At the room temperature of 300 K the transition is not smooth, with many oscillations of the average magnetization and individual spins vibrations throughout the entire reversal process. The exchange interaction competes actively with the thermal agitation introduced by the thermal field. First neighboring spins struggle to be very parallel between each other, nonetheless the behavior of the NP magnetization dynamics is that of the expected single domain particle.
